# Associations between occupation exposure to Formaldehyde and semen quality, a primary study

**DOI:** 10.1038/srep15874

**Published:** 2015-10-30

**Authors:** Hai-xu Wang, He-cheng Li, Mo-qi Lv, Dang-xia Zhou, Li-zhi Bai, Liang-zhi Du, Xia Xue, Pu Lin, Shu-dong Qiu

**Affiliations:** 1 Department of Pathology, School of basic medical sciences, Health science center, Xi’an Jiaotong University, Xi’an, 710061, China; 2 Reproductive Medicine Center, Medical School, Xi’an Jiaotong University, Xi’an, 710061, China; 3 Reproductive Medical Center, Department of Obstetrics and Gynecology, Xijing Hospital of the Fourth Military Medical University, Xi’an, 710032, China; 4 Key Laboratory of Environment and Genes Related to Diseases, Ministry of Education, Xi’an, 710061, China; 5 Department of Urinary Surgery, The Second Affiliated Hospital, Xi’an Jiaotong University, Xi’an, 710004, China; 6 Reproductive Medicine Center, Maternal and child care Hospital of Shaanxi Province, Xi’an, 710003, China; 7 Electric Power Science Research Institute of Shaanxi Province, Xi’an, 710054, China

## Abstract

Formaldehyde (FA), a ubiquitous environmental pollutant, has long been suspected of having male reproductive toxicity. However, FA male reproductive toxicity was inconclusive due to dearth of human studies. Therefore, we sought to investigate whether occupational exposure to FA affects semen quality. Semen quality including five conventional parameters and seven kinematics parameters were compared between 114 male workers occupationally exposed to FA and 76 referents. FA exposure index (FEI) was measured and calculated. Our results showed that sperm progressive motility, total sperm motility, VCL, VSL and VAP were statistically significant decreased in FA exposure workers compared with the referents. Moreover, FEI was significantly negative associated with sperm progressive motility (β = −0.19, *P* = 0.01) and total sperm motility (β = −0.23, P = 0.004). In addition, a significant elevated risk of abnormal sperm progressive motility were observed in both low- (OR = 2.58; 95% CI: 1.11–5.97) and high-FA-exposed group (OR = 3.41; 95% CI: 1.45–7.92) respectively. Furthermore, a significant increased risk was also estimated for abnormal total sperm motility in both low- (OR = 3.21; 95% CI: 1.24–8.28) and high-FA-exposed group (OR = 4.84; 95% CI: 1.83–12.81) respectively. In conclusion, our study revealed the adverse effects of FA occupation exposure on semen quality, especially on sperm motion parameters.

Decline in fertility is a growing concern and it has become an important public health issue in recent years. Studies demonstrated that the fertility rate in the USA fell 45% between 1960 and 2002^1^. Moreover, around 14% of couples in industrialized countries experience difficulty with conception at some point in their lives^2^. Male factors account for nearly half of the infertility cases^3^, unfortunately, a high proportion of male infertility are unexplained or idiopathic^3^,^4^. Concurrently, researchers have reported a worldwide decline in human semen quality during the last 50 years^5^,^6^,^7^,^8^. With the increasing of environmental contaminant in recent years, there has been elevated awareness of the potential risks of environmental factors on male reproduction^9^,^10^.

Formaldehyde (FA), the recently classified carcinogen and ubiquitous environmental contaminant, is widely used in resins, construction, wood processing, textiles, hospitals, laboratories and chemical industry for its preserving, sterilizing and stabilizing properties^11^. As an important chemical for global economy, FA output has reached more than 46 billion pounds annually worldwide. Over the last 20 years, China has experienced rapid economic growth and a simultaneous rise in demand for FA. In 2004, China surpassed the United States as the largest FA producer and consumer in the world. China’s actual FA output has reached a staggering 12000 kt in 2007, about 4000 times the amount five decades earlier^12^. Coinciding with the growing production and consumption, FA pollution has also increased considerably in recent years. Emerging evidence showed an association between FA exposure and multiple adverse health effects such as asthma, nasopharyngeal cancer and probably leukemia etc.^13^,^14^,^15^.

Recently, it has also been reported that FA gas exposure are detrimental to male fertility in many animal experiments^16^,^17^,^18^,^19^,^20^,^21^,^22^,^23^. Ozen *et al.* (2005)^16^ reported that FA gas inhalation at dose of 5 and 10 ppm damaged spermatogenetic cells and increased Hsp 70 synthesis in rats. Liu *et al.* (2009)^17^ demonstrated that FA gas exposure at dose of 20–200 mg/m^3^ induced the damage of spermatogenetic cells and expanded simple tandem repeats (ESTR) mutations in mice. Kose *et al.* (2012)^18^ concluded that the testosterone levels, the epididymal sperm concentration and the progressive sperm motility significantly decreased in rats exposed to FA vapor (10 ppm/1 h) for 35 days. Vosoughi *et al.* (2013)^19^ disclosed that FA vapor (10 and 20 ppm) exposure destroyed testicular structure and decreased sperm concentration, viability, normal morphology, and progressive motility, in addition to increasing the percentage of immotile sperm. Moreover, our earlier studies^20^,^21^,^22^,^23^ also showed FA exposure damaged testicular and epididymal histological morphology, and induced oxidative stress in rats exposed to FA gas at dose of 2.46–10 mg/m^3^.

Despite evidence in animals experiment has accumulated in support of an association between harmful male reproductive toxicity and FA exposure, however, very few human epidemiological studies have explored the relationship between FA exposure and male reproductive outcomes. A Finnish cohort study^24^ observed the sperm count, morphology of paternal exposure to FA in 11 autopsy service workers and 11 matched controls. Although reduced sperm count correlated with increased abnormal morphology was observed in the exposed male workers but not in the control. However, as acknowledged by the authors, given the small size of the exposure groups (n = 11 in each group), the study had very low statistical power. Another study^25^ focused on risk of spontaneous abortion (SAB) resulting from paternal exposure to FA in Finland. In a cohort of 596 pregnancies, their results showed no overall excess of SAB in women whose husbands were exposed to FA. Additionally, our previous study evaluated the effects of paternal occupational exposure to FA on reproductive outcomes in a Chinese population. We found that 2.8 times increased risk of prolonged time to pregnancy and 1.9 times elevated risk of spontaneous abortion in wives of male workers occupationally exposed to FA when compared with the controls^26^. As described above, to date, there have been very limited human studies on the affection of FA exposure to reproductive toxicity. Besides, the small sizes of cases, as well as the self-reported nature of exposure and outcome variables, may hamper interpretation of the results. Therefore, the present study was designed to investigate potential associations between the FA exposure levels and semen quality in male workers occupationally exposed to FA. Our efforts should add some new objective evidence for the hypothesis that FA occupation exposure has adverse effects on male reproductive health.

## Materials and Methods

### Subject recruitment

The experimental protocols were approved by the Institutional Medical Ethics Committee of Xi’an Jiaotong University. The experiment methods were carried out in accordance with the approved guidelines. A recruitment campaign to enroll participants from seven wood processing industries (plywood production) was organized by Pathology Department of Xi’an Jiaotong Unversity. The purpose of this study was informed to all subjects, and written informed consent forms were obtained from all subjects.

Inclusion criteria for FA exposure group were as follows: men aged from 23 to 40 years old at the time of inclusion; Chinese Han ethnicity; and men who had worked in the FA exposure environment for at least 24 months. On the other hand, men who had lived in a newly built or recently decorated house; men who had genital malformations or other chronic diseases were excluded form the study. At the same time, age-matched male Han population volunteers who had lived in same place of residence were chosen as reference group with respect to educational level and socioeconomic status. For the reference group, we only selected those who exposure to FA or other reproductive toxicants was avoided. The reference group mainly consisted of salesmen and clerks.

### Questionnaire

According to the methods used in our previous study^26^, a semi-structured interview questionnaire was completed by all participants, including baseline information about sociodemographics (age, nationality, education level, and income), lifestyle (e.g. smoking status and alcohol consumption), abstinence duration, previous or current diseases, and occupational exposure (time and duration of occupational exposure). All participants claimed that their lifestyles and environments had not changed for at least 6 months prior to semen collection.

### Physical examination

A physical examination, including height and weight, was performed. Body mass index (BMI) was calculated for every subjects. In addition, two infertility specialists performed the genital examination of all subjects; the possible presence of genital malformations and diseases was investigated.

### FA exposure Assessment

According to the method of our previous study, with minor modification^26^, FA exposure index (FEI) was measured and calculated for every worker. Firstly, the workers were asked to give information about their workplace, work tasks, work duration and time. Secondly, the FA concentration in the workplace of each worker was measured by stationary sampling on the day when the investigation was done. In detail, the formaldehyde detector (4160-2; Interscan, Chatsworth, CA) was stable for about 15 minutes in the environment before the zero adjustment. Next zero was set at the sampling mode, and then removed off the filter. The stable reading on the screen is the on-site FA concentration. The total sampling time in single measurement lasts about 25 minutes, and we monitored the FA concentration at three different time points (9:00 AM, 12:00 AM and 15:00 PM, respectively) during one workday. The mean value of the three measurements was used as FA exposure concentration of the worker. Due to the limitations of the measurement time points and frequency, no data were available for potential short-term exposure peaks. Finally, FEI was calculated as follows: FA exposure index (FEI) = concentration of FA (mean value of three measurements, in mg/m^3^) × exposed work time during a workday × exposure duration (year). According to the FEI level, two exposure classes were further defined so that the number of men in the low and high FA-exposed groups had the ratio of 1:1.

### Semen collection and analysis

All subjects were not taken any medical or surgical treatments in the three months prior to the semen examination. The semen analysis was performed within 2 weeks after the FA exposure measurements. Subjects were requested to provide a semen sampling after 2–7 days of abstinence. Semen samples were collected by masturbation in a sterile wide-mouthed calibrated container and semen were immediately analyzed within 60 min after collection. All the semen analyses were conducted by two well-trained technicians using the same apparatus in a blinded manner, in order to reduce the variance of assessment of sperm characteristics.

Semen analyses were performed using a computer-automated semen analysis system (CASA) (WLJY-9000, China). According to the manufacturer’s guidelines, freshly collected semen samples were allowed to liquefy for 20 min at 37 °C. Conventional semen parameters included semen volume, sperm concentration, total sperm count, sperm progressive motility and total sperm motility was assessed. Moreover, the seven kinematic parameters were detected according to the WHO laboratory manual (2010)^27^. Of which, Curvilinear velocity (VCL) is the average velocity measured over the actual point-to-point track followed by the cell. Straight line velocity (VSL) is the time-average velocity of a sperm head along the straight line between its first and last detected positions. Linearity (LIN) is the linearity of the curvilinear trajectory calculated as VSL/VCL × 100. The time-average velocity (VAP) measures the sperm head along its spatial average trajectory (i.e. smoothed version of VCL). Straightness (STR) is the LIN of the sperm average path calculated as VSL/VAP × 100. Mean angular displacement (MAD) is the time-averaged absolute values of the instantaneous turning angle of the sperm head along its curvilinear trajectory. Amplitude of lateral head displacement (ALH) is the maximum lateral displacement of a sperm head about its spatial average trajectory.

### Statistical analysis

At first, a descriptive analysis was performed following data collection. Means and standard deviations were calculated for continuous variables with checking for normality. Frequency was evaluated for the different categorical variables. Moreover, because conventional semen parameters follow markedly skewed (non-normal) distribution, the 25th, median and 75th percentiles were computed.

Secondly, sociodemographic characteristics and semen parameters were compared between the FA exposure groups and the referent group by using *t*-test or One-way ANOVA for normally distributed continuous variables, Kruskal–Wallis test for non-normally distributed continuous variables, and Chi-Square-Fisher’s exact test for categorical variables respectively.

Thirdly, we used multiple linear regression models to estimate the associations between FA exposure and semen parameters. Semen parameters were log-normal (In) transformed to improve the normality as dependent variables in the linear models. In addition, logistic regression analysis was used to further calculate the crude and adjusted odds ratios (ORs) of abnormal semen parameters with 95% confidence intervals (CIs). A full model that included all possible confounding factors to be examined in regression was used. Selection of confounding factors for the final model was based on their importance in the literature and biological plausibility^28^,^29^,^30^, the variables entered into the regression model included age, body mass index, education, income, smoking, drinking and abstinence duration.

All statistical analyses were carried out using SPSS statistical software version 16.0 (SPSS, Chicago, USA), and *P* < 0.05 was considered statistically significant.

## Results

### Subjects characteristics

Totally 124 (62.3%) male worker occupationally exposed to FA were recruited and eligible for the study whereas 75 workers refused to provide semen. However, in the phase of semen collection, 8 workers were excluded for failing to collect their semen samples and 2 workers were excluded for spillage of the sample semen. Finally, a total of 114 male worker occupationally exposed to FA were eligible and completed all the steps of the study.

On the other hand, 81 referents (40.5%) were recruited and screened for the study. Of these, 3 referents were excluded for failing to collect the semen samples and 2 were excluded for spillage of the sample semen. Finally, a total of 76 eligible referents participated in this study.

By interviewing, the participants in the FA exposure and reference group were not engaged in heavy physical labor, and didn’t work and live in high temperature environment. In addition, the participants didn’t experience extra mental stresses in both groups. Table 1 shows the sociodemographic characteristics and abstinence duration of FA exposure workers and referents. Compared with the referents, the FA- exposed subjects were similar in age, body mass index (BMI), education level, income, cigarette intake, alcohol consumption and duration of abstinence. The mean age in both groups was around 30, the average body mass index (BMI) was about 24, and the majority of the subjects had completed primary education. The average monthly income in both groups was around 1800 RMB. More than half of the subjects used tobacco and alcohol in both groups. In addition, the mean duration of abstinence was about 4 days in both FA exposure group and reference group.

### FA exposure level

FA concentrations in 76 referents ranged from 0.0 to 0.02 mg/m^3^. These values can be neglected because they were far lower than FA occupation exposure limit. Moreover, the concentrations of FA measured in air ranged from 0.22 to 2.91 mg/m^3^ in all 114 workers. The FA exposure index (FEI) calculated in each FA-exposed workers ranged from 4.54 to 195.08. We further divided the workers into low and high-FA exposed groups in accordance with the median level (56.55) of FEI so that the number of workers in two FA-exposed groups had the ratio of 1:1.

### Semen parameters

The outcome of semen conventional parameters was summarized in Table 2. No statistically significant differences were found in semen volume, sperm concentration, total sperm count between FA exposure groups and reference group. However, the results clearly suggest that there has been a statistically significant decrease in sperm progressive motility (38.6% and 36.0% vs. 49.9%) and sperm total motility (49.8% and 43.8% vs. 59.5%) in low- and high-FA exposure groups when compared with reference group. Moreover, these reductions are in a dose-dependent trend.

In addition, the data of seven semen kinematic parameters were reported in Table 3. There were no significant differences in terms of LIN, STR, MAD and ALH between FA exposure group and reference group. However, there was statistically significant decrease in VCL, VSL and VAP in FA exposure groups when compared with reference group, where a dose-dependent trend was also observed.

### Associations between FA exposure and semen quality

To clarify the effects of FA exposure on semen quality, the associations between FA exposure index (FEI) and semen parameters (conventional and kinematic parameters) was calculated by multiple linear regression and the results were summarized in Table 4 and 5 respectively. After adjusting for confounding factors, FEI showed significantly negative associations with sperm progressive motility (β = −0.19, *P* = 0.01) and total sperm motility (β = −0.23, *P* = 0.004), respectively.

Table 6 provides information on crude and adjusted ORs for the risks between abnormal (below-normal values) semen parameters and FA occupational exposure. After correction for confounding factors, a significant elevated risk of abnormal sperm progressive motility were found in low-FA-exposed group (OR = 2.58; 95% CI: 1.11–5.97) and high-FA-exposed group (OR = 3.41; 95% CI: 1.45–7.92) respectively compared with the reference group. Moreover, a significant increased risk was also observed for abnormal total sperm motility in low-FA-exposed group (OR = 3.21; 95% CI: 1.24–8.28) and high-FA-exposed group (OR = 4.84; 95% CI: 1.83–12.81) respectively when compared with the reference group after correction for confounding factors. The coefficients (OR) with CIs and p-values of the other 7 variables (age, cigarette intake, alcohol consumption, abstinence duration, body mass index, education, and income) belonging to the models were demonstrated in Supplementary Table 4, Supplementary Table 5 and Supplementary Table 6 respectively.

In addition, slightly increased risks of abnormal semen volume, sperm concentration and total sperm count was found in FA exposure groups, but this association was not reached statistical significance even after adjusting for confounding factors (Table 6).

## Discussion

Our study is the first to reveal the adverse effects of FA occupation exposure on semen quality parameters in a Chinese population. Our study found that semen parameters included sperm progressive motility, total sperm motility, VCL, VSL and VAP were statistically significant decreased in FA exposure groups at a dose-dependent trend compared with reference group. Moreover, personal FEI (FA exposure index) showed significantly negative associated with sperm progressive motility (β = −0.19, *P* = 0.01) and sperm total motility (β = −0.23, *P* = 0.004) after adjusting for confounding factors. In addition, compared with the reference group, a significant elevated risk of abnormal sperm progressive motility were observed in both low- (OR = 2.58; 95% CI: 1.11–5.97) and high-FA-exposed group (OR = 3.41; 95% CI: 1.45–7.92), respectively, after correction for confounding factors. Furthermore, a significant increased risk was also estimated for abnormal total sperm motility in both low- (OR = 3.21; 95% CI: 1.24–8.28) and high-FA-exposed group (OR = 4.84; 95% CI: 1.83–12.81) after correction for confounding factors.

During the last few decades a possible degradation in human semen quality has been studied intensively and many studies have suggested the declining trend of sperm quality worldwide^5^,^6^,^7^,^8^. With increased evidence of a decline in semen quality in recent years, public, government and scientific concerns about the effects of environmental changes on male reproductive health have grown to become a major preoccupation in many countries^31^,^32^. As one of the ubiquitous environmental contaminant in the world, although preventive measures aimed at reducing FA contaminant have been implemented, exposure to FA remains one of the most prominent environmental health problems^33^,^34^. The detrimental effects of FA on male reproduction have also been shown in many animal experiments^16^,^17^,^18^,^19^,^20^,^21^,^22^,^23^. However, due to species differences, the result cannot be directly extrapolated to humans. FA male reproductive toxicity was inconclusive due to the scare of detailed data in human studies. Our present results suggested the relationships between FA exposures and human sperm quality parameters. Our findings further increase the objective evidence to support the hypothesis that FA exposure has adverse effect on male reproduction.

There is a widespread human exposure to FA, the principal exposure route in environment is through inhalation. Historically, occupational exposure has been the dominant source of FA exposure in China^33^. Current Occupational Exposure Limit (OEL) for FA^35^ in China is 0.5 mg/m^3^, and some countries employ time-weighted averages (TWA) and/or short-term exposure limits (STEL). For example, the USA’s OEL^36^ is 0.75ppm (0.92 mg/m^3^, 8-h TWA) with a STEL of 2ppm (2.46 mg/m^3^), while the United Kingdom^37^ is 2ppm (2.46 mg/m^3^) for both TWA and STEL. Although the low OEL for FA in China compared with other countries, FA exposure levels have been high across many different Chinese industries. For example, Wong *et al.*^38^ reported that the average FA concentration in the wood processing industry was 3.07 ± 5.83 mg/m^3^. Fan *et al.*^39^ found the FA concentrations in several hospital pathology laboratories were as high as 5.84 mg/m^3^. The investigation of Zhang *et al.*^40^ showed that the FA concentration in some anatomy laboratories was above 10 mg/m^3^. In the present study, we found that the concentrations of FA measured in air ranged from 0.22 to 2.91 mg/m^3^ in 114 workers of wood processing industry, among of them, part FA concentrations are far higher than OEL standards in China (0.5 mg/m^3^). We speculated that the wood processing industry has the high FA concentration, which is caused in part by unventilated workshops and a lack of employee safety precautions.

Semen quality is one of the most valuable indications of male reproductive health, and it is associated with fertility status^29^,^41^. In our study, semen quality parameters included both semen conventional parameters and kinematic parameters were all analyzed by Computer-Assisted Sperm Analysis (CASA). CASA not only decreases the time spended in sperm observation, but reduces intra-observer differences and improve the accuracy of final results. CASA method^42^ makes the assessment of semen quality more objective, detailed and reproducible.

In our study, semen volume, sperm concentration and total sperm count were not markedly declined in FA occupational exposure groups. However, semen motion parameters included sperm progressive motility, total sperm motility, VCL, VSL and VAP were statistically significant decreased in FA exposure groups compared with the reference. Our results indicated sperm motility might be more sensitive to environmental FA exposure than other semen parameters. Our present findings is not consistent with the Finland study (only 11 autopsy worker) that reported that no effects on sperm were seen from FA or its metabolites in this population after occupational exposure^24^. However, the decline of the motility parameters due to FA exposure has been highlighted in several animal experiments which found FA caused regressive histological changes in the seminiferous tubules and epididymis resulting in the suppression of sperm maturation and motility^18^,^19^,^21^.

Sperm motility is one of the most important indicators in assessing sperm quality^41^, it is also an important indicator for sperm fertilization. The decline in the sperm motion parameters may reflect decreased sperm functions that allow the sperm to reach the oocyte to complete fertilization^43^. Hirano *et al.* (2001)^44^ reported that VCL, VSL, VAP have been correlated with fertilization rates *in vivo* and may be bioindicators of the fertilizing ability of human sperm. Our earlier study^26^ found that the wives of male workers occupationally exposed to FA have increased risk of prolonged TTP (Time-to-pregnancy) and spontaneous abortion. Together with our current findings, we speculated adverse reproductive outcomes such as prolonged TTP and spontaneous abortion might be attributed to the decline of sperm motion ability. Some studies showed strong evidence that FA itself does not reach systemic circulation^45^,^46^. The pathogenic effect of FA exposure on sperm motility might be a result of free radical-generated oxidative stress and a distriburbance of the redox equilibrium^20^,^21^,^22^,^23^,^47^. Oxidative stress promotes a dose-dependent increase in tyrosine nitration and S-glutathionylation and alters motility and the ability of spermatozoa to undergo capacitation^48^. However, the underlying molecular mechanisms of FA exposure on sperm were still remaining unclear.

Moreover, in this study, we use FA exposure index (FEI) to reflect FA exposure level. FEI not only reflect FA exposure concentration, but also combined with exposure duration. This indicator is more precise to reflect the actual FA occupational exposure level. Our results showed that FEI was significantly negative associated with semen motion parameters included sperm progressive motility (β = −0.19, *P* = 0.01) and sperm total motility (β = −0.23, *P* = 0.004). That is, a 10-fold increase in FEI was found to be associated with a 1.9-fold decrease in sperm progressive motility, and a 2.3-fold decrease in total sperm motility. In addition, compared with the referent, a significant elevated risk of abnormal sperm progressive motility were observed in both low- (OR = 2.58; 95% CI: 1.11–5.97) and high-FA-exposed group (OR = 3.41; 95% CI: 1.45–7.92) respectively. Furthermore, a significant increased risk of abnormal total sperm motility in both low- (OR = 3.21; 95% CI: 1.24–8.28) and high-FA-exposed group (OR = 4.84; 95% CI: 1.83–12.81) were also detected. Our results further demonstrated the male reproductive toxicity of FA exposure might be dose-dependent.

In this study, we applied some methods to overcome the potential bias in different phases of this study. At first, in the phase of subject recruitment, strict inclusion criteria and exclusion criteria were used to screen subjects. Except the similar ethnic origin, we recruited only those subjects who had worked in the FA exposure environment for at least 24 months. Since too short exposure time was not suitable to evaluate the association between FA occupational exposure and reproductive health. In addition, modern home building and furnishings are the main sources of indoor FA pollution globally, newly built residences and recently remodeled apartment release high levels of indoor FA. Therefore, to reduce the influence of family environmental FA pollution, we excluded the subjects who live in a newly built residences and recently remodeled apartment. On the other hand, to decrease the selection bias, we chose reference group with respect to similar educational level, socioeconomic status and residential environment. Secondly, in the process of FA exposure assessment, we measure the air concentration of FA in occupational environment and calculate FA exposure index (FEI) according to exposure duration and concentration. This approach relies on personal work area monitoring data to construct an exposure assessment of FA, it is more precise to reflect the true FA occupational exposure level. Thus, it reduces the potential for exposure misclassification. Thirdly, in the process of semen analysis, semen quality parameters included both semen conventional parameters and kinematic parameters were all analyzed by Computer-Assisted Sperm Analysis (CASA) by two well-trained technicians. CASA method makes the semen assessment more objective, detailed and reproducible^42^.

Although we used many methods to reduce bias, we realize that our study was not without limitations. A potential limitation of this study was the fact that only FA concentration was measured and evaluated for each subject. In the wood processing industry, except mainly exposure to FA, there may be exposure to other organic solvents such as phenols and wood preservatives^49^. Phenol has been reported to have genotoxicity and induced sister chromatid exchange in human cells^50^. In addition, occupational exposure to organic solvents has been related to low motile sperm count in men^51^. Therefore, in the future study the concentration of other organic solvents should be monitored and calculated.

## Conclusions

The present study suggested the adverse effects of FA occupation exposure on semen quality, especially on sperm motion parameters in a dose-dependent trend, which might increase the objective evidence to support the hypothesis that FA exposure has negative effects on male reproduction. Given the importance of human reproductive health and the current wide usage of FA, it is valuable to further investigate the correlations between FA exposure and semen quality in a large cohort.

## Additional Information

**How to cite this article**: Wang, H.-x. in *et al.* Associations between occupation exposure to Formaldehyde and semen quality, a primary study. *Sci. Rep.* **5**, 15874; doi: 10.1038/srep15874 (2015).

## Supplementary Material

supplementary tables

## Figures and Tables

**Table 1:** Comparisons of Sociodemographic characteristics and Abstinence Duration between Formaldehyde occupational exposure group and reference group.

Characteristics	Reference group (n = 76)	FA exposure group (n = 114)	*P*
Age (years)	29.26 ± 3.51	29.84 ± 4.06	0.293
Employment duration (years)	5.37 ± 1.83	6.11 ± 1.45	0.132
BMI (kg/m^2^)	23.88 ± 1.32	24.09 ± 1.78	0.617
Education level			0.174
Low(<6 years)	19(23.5%)	40(32.2%)	
High(≧6 years)	62(76.5%)	84(67.8%)	
Income (RMB/per month)	1814.20 ± 680.59	1733.87 ± 608.86	0.379
Cigarette intake			0.348
Low (no smoking)	13(16.1%)	15(12.1%)	
Moderate(<10 Cigarettes/day)	63(77.8%)	105(84.7%)	
High (≧10 Cigarettes/day)	5(6.1%)	4(3.2%)	
Alcohol consumption			0.709
Low (no drinking)	38(39.3%)	46(36.1%)	
Moderate(<1000g/day)	56(57.6%)	73(58.3%)	
High (≧1000g/day)	3(3.1%)	7(5.6%)	
Duration of abstinence	3.97 ± 1.51	4.13 ± 1.59	0.930

BMI: Body mass index.

**Table 2:** Comparisons of semen conventional parameters between FA occupational exposure groups and reference group.

Characteristics	Reference group (n = 76)	Low FA-exposed group(n = 57)	High FA-exposed group(n = 57)	*P*-value^a^
Semen volume (ml)	2.5(2.0–3.6)	2.7(1.6–3.8)	2.3(2.0–3.5)	0.451
Sperm concentration (10^6^/ml)	52.2(35.0–76.9)	52.1(31.6–76.8)	54.9(34.7–69.3)	0.880
Total sperm count (10^6^)	134.3(83.1–215.7)	121.6 (76.6–211.9)	126.3(71.8–184.3)	0.648
Sperm progressive motility (PR,%)	49.9(35.3–63.1)	38.6(28.5–46.6)	36.0(25.5–45.6)	<0.001*
Total motility (PR + NP,%)	59.5(46.0–72.4)	49.8(36.7–60.4)	43.8(32.7–53.1)	<0.001*

Note: Data are expressed as median values (interquartile ranges 25th–75th percentile shown in parentheses).

PR: sperm progressive motility equals the spermatozoa moving actively, either linearly or in a large circle, regardless of speed.

NP: non-progressive motility equals all the other patterns of motility with an absence of progression.

^a^Kruskal–Wallis analysis of variance was used to compare the median between groups *P < 0.05.

**Table 3:** Comparisons of semen kinematic parameters between FA occupational exposure groups and reference group.

Characteristics	Reference group (n = 76)	Low FA-exposed group (n = 57)	High FA-exposed group (n = 57)	*P*-value^b^
VCL(μm/s)	52.31 ± 10.02	48.87 ± 9.64^a^	47.16 ± 10.15^a^	0.010*
VSL(μm/s)	35.48 ± 8.12	32.08 ± 7.35^a^	31.16 ± 7.34^a^	0.003*
LIN(VSL/VCL)	66.25 ± 7.84	63.88 ± 9.98	65.15 ± 6.61	0.263
VAP(μm/s)	38.56 ± 8.24	35.07 ± 7.25^a^	33.96 ± 7.52^a^	0.002*
STR(VSL/VAP)	88.64 ± 3.80	87.27 ± 4.61	88.27 ± 3.77	0.149
MAD(°)	53.44 ± 7.44	54.73 ± 9.59	53.53 ± 7.19	0.618
ALH(μm)	3.46 ± 1.11	3.44 ± 1.24	3.41 ± 1.03	0.979

VCL(μm/s), curvilinear velocity; VSL(μm/s), straight-line velocity; LIN(VSL/VCL), linearity; VAP(μm/s), average path velocity; STR(VSL/VAP), straightness; MAD(°), mean angular displacement; ALH(μm), amplitude of lateral head displacement.

^a^When compared with the reference group *P* < 0.05.

^b^One-way ANOVA was used to compare the mean between groups *P *<* 0.05.

**Table 4:** Associations of Formaldehyde exposure index (FEI) with semen conventional parameters before and after adjusting for confounding factors.

Formaldehyde exposure	Volume (ml)^a^	Concentration(10^6^/ml)^a^	Total sperm count(10^6^)^a^	Sperm progressive motility (PR,%)^b^	Total motility (PR + NP,%)^b^
**Crude** β coefficients	−0.02	−0.05	−0.27	−0.17	−0.21
95% CI	(−0.07,0.02)	(−0.20,0.10)	(−0.72,0.18)	(−0.23,−0.11)	(−0.27,−0.14)
*P*-Value	0.33	0.54	0.24	0.02*	0.01*
**Adjusted** β coefficients	−0.02	−0.02	−0.20	−0.19	−0.23
95% CI	(−0.08,0.03)	(−0.19,0.14)	(−0.68,0.29)	(−0.25,−0.12)	(−0.30,−0.16)
*P*-Value	0.43	0.77	0.38	0.01*	0.004*

PR: sperm progressive motility equals the spermatozoa moving actively, either linearly or in a large circle, regardless of speed.

NP: non−progressive motility equals all the other patterns of motility with an absence of progression.

Regression coefficients were adjusted for age, cigarette intake, alcohol consumption, body mass index, income, education and abstinence duration.

^a^Result expressed as the relative percent change for volume, concentration, total sperm count. This number is converted from the antilog of the regression coefficient (β) of the log−linear model.

^b^For sperm progressive motility and total motility, result expressed as the absolute change. *P < 0.05.

**Table 5:** Associations of Formaldehyde exposure index (FEI) with semen kinematic parameters before and after adjusting for confounding factors.

	VCL(μm/s)	VSL(μm/s)	LIN(VCL/VSL)	VAP(μm/s)	STR(VSL/VAP)	MAD(°)	ALH(μm)
**Crude** β coefficients	−0.07	−0.05	0.001	−0.05	0.002	−0.01	−0.003
95% CI	(−0.15,0.03)	(−0.12,0.02)	(−0.04,0.04)	(−0.12,0.02)	(−0.02,0.02)	(−0.05,0.03)	(−0.008,0.002)
*P*-Value	0.27	0.31	0.95	0.39	0.98	0.55	0.22
**Adjusted** β coefficients	−0.08	−0.05	0.002	−0.05	0.004	−0.01	−0.004
95% CI	(−0.18,0.04)	(−0.11,0.01)	(−0.04,0.04)	(−0.13,0.02)	(−0.02,0.03)	(−0.05,0.02)	(−0.010,0.001)
*P*-Value	0.15	0.36	0.92	0.31	0.67	0.51	0.20

Regression coefficients were adjusted for age, cigarette intake, alcohol consumption, body mass index, income, education and abstinence duration.

**Table 6:** ORs and 95% CI for below-normal values of semen parameters associated with FA occupational exposure before and after adjusting for confounding factors.

	Reference group(n = 76)	Low-FA-exposed group (n = 57)		High-FA-exposed group (n = 57)	
		crude	adjusted	crude	adjusted
Semen volume (<1.5 ml) *P*	Ref	0.16	0.27	0.06	0.15
OR(95% CI)	1.0	2.10(0.75–5.90)	1.83(0.63–5.36)	2.63(0.96–7.18)	2.28(0.75–6.91)
Sperm concentration (<15 × 10^6^/ml) *P*	Ref	0.44	0.54	0.72	0.81
OR(95% CI)	1.0	1.84 (0.39–8.55)	1.67(0.33–8.43)	1.35(0.26–6.96)	1.25(0.21–7.35)
Total sperm count (<39 × 10^6^)*P*	Ref	0.42	0.47	0.16	0.18
OR(95% CI)	1.0	1.67(0.48–5.77)	1.59(0.45–5.61)	2.32(0.72–7.51)	1.73(0.49–6.15)
Sperm progressive motility (PR,%<32%) *P*	Ref	0.02*	0.03*	0.001*	0.005*
OR(95% CI)	1.0	2.67(1.17–6.10)	**2.58(1.11–5.97)**	3.88(1.73–8.72)	**3.41(1.45–7.92)**
Total motility (PR + NP,%<40%) *P*	Ref	0.01*	0.02*	0.001*	0.001*
OR(95% CI)	1.0	3.32(1.31–8.43)	**3.21(1.24–8.28)**	4.96(2.00–12.31)	**4.84(1.83-12.81)**

OR: odd ratio; CI: confidence interval.

Note: abnormal sperm parameters were defined by the World Health Organization (WHO, 2010) standards: semen volume <1.5 ml, sperm concentration <15 × 10^6^/ml, total sperm count <39 × 10^6^/ml, total sperm motility <40%, and progressive motility <32%.

Regression coefficients were adjusted for age, cigarette intake, alcohol consumption, body mass index, income, education and abstinence duration.

*P < 0.05.
