# Supplementary material for: Associations between occupation exposure to Formaldehyde and semen quality, a primary study
Source: Sci Rep. 2015 Oct 30;5:15874. doi: 10.1038/srep15874 (PMC4626826; doi:10.1038/srep15874)
Supplement: supplementary tables [file srep15874-s1.doc]

Associations between occupation exposure to Formaldehyde and semen quality, a primary study

**Hai-xu Wang^1, 2, 3, #^, He-cheng Li ^2, 5, #^, Mo-qi Lv^1, 2^, Dang-xia Zhou^1,2,4, *^, Li-zhi Bai^1,4^, Liang-zhi Du^1,4^, Xia Xue^6^, Pu Lin^7^, Shu-dong Qiu^2^**

*^1^Department of Pathology, Medical School, Xi’an Jiaotong University, Xi’an, 710061, China*

*^2^Reproductive Medicine Center, Medical School, Xi’an Jiaotong University, Xi’an, 710061, China ^3^Reproductive Center, The Fourth Military Medical University, Xi’an, 710032, China*

*^4^Key Laboratory of Environment and Genes Related to Diseases, Ministry of Education, Xi’an, 710061, China*

*^5^Department of Urinary Surgery, The Second Affilated Hospital,, Xi’an Jiaotong University, Xi’an, 710004, China*

*^6^Reproductive Medicine Center, Maternal and child care Hospital of Shaanxi Province, Xi’an, 710003, China*

*^7^Electric Power Science Research Institute of Shaanxi Province, Xi’an, 710054, China*

^#^Authors contributed equally to this work.

***Correspondence to**

Dang-xia Zhou, Pathology department, Medical School, Xi’an Jiaotong University, Xi’an, 710061, China.

Tel.: +86 2982655189-8008

E-mail address: [zhoudx2010@163.com](mailto:zhoudx2010@163.com)

**Table 4-supplement**  Associations of Formaldehyde exposure index (FEI) with semen conventional parameters before and after adjusting for confounding factors ^c^

|  | Volume (ml)^a^ | Concentration  (10^6^/ml)^a^ | Total sperm count(10^6^) ^a^ | Sperm progressive motility (PR,%) ^b^ | Total motility (PR+NP,%) ^b^ |
| --- | --- | --- | --- | --- | --- |
| **Crude β coefficients** | -0.02 | -0.05 | -0.27 | -0.17 | -0.21 |
| 95%CI | (-0.07,0.02) | (-0.20, 0.10) | (-0.72,0.18) | (-0.23,-0.11) | (-0.27,-0.14) |
| *P*-Value | 0.33 | 0.54 | 0.24 | **0.02*** | **0.01*** |
| **Adjusted β coefficients ^d^** | -0.02 | -0.02 | -0.20 | -0.19 | -0.23 |
| 95%CI | (-0.08,0.03) | (-0.19, 0.14) | (-0.68,0.29) | (-0.25,-0.12) | (-0.30,-0.16) |
| *P*-Value | 0.43 | 0.77 | 0.38 | **0.01*** | **0.004*** |
| **Age** | | | | | |
| β coefficients | -0.03 | 0.05 | 0.06 | -0.53 | -0.71 |
| 95%CI | (-0.09,0.03) | (-0.02,0.12) | (-0.03,0.15) | (-2.17,1.11) | (-2.82,1.40) |
| *P*-Value | 0.36 | 0.25 | 0.14 | 0.43 | 0.24 |
| **Cigarette intake** | | | | | |
| β coefficients | -0.04 | 0.16 | -0.08 | -1.12 | -1.28 |
| 95%CI | (-0.10,0.02) | (-0.08,0.40) | (-0.19,0.03) | (-8.18,5.95) | (-5.68,3.12) |
| *P*-Value | 0.23 | 0.07 | 0.46 | 0.35 | 0.23 |
| **Alcohol consumption** | | | | | |
| β coefficients | -0.12 | -0.08 | 0.14 | 1.12 | 1.29 |
| 95%CI | (-0.26,0.02) | (-0.23,0.07) | (-0.04,0.32) | (-3.16,5.41) | (-2.76,5.34) |
| *P*-Value | 0.14 | 0.23 | 0.11 | 0.31 | 0.15 |
| **Abstinence duration** | | | | | |
| β coefficients | 0.08 | 0.12 | 0.11 | -1.48 | -2.62 |
| 95%CI | (-0.05,0.21) | (-0.06,0.30) | (-0.07,0.29) | (-2.25,-0.71) | (-3.83,-1.41) |
| *P*-Value | 0.21 | 0.16 | 0.17 | **0.04*** | **0.02*** |
| **BMI** | | | | | |
| β coefficients | -0.05 | -0.02 | -0.07 | 1.50 | 1.13 |
| 95%CI | (-0.12,0.02) | (-0.05,0.01) | (-0.18,0.05) | (-1.13,4.13) | (-0.42,2.68) |
| *P*-Value | 0.20 | 0.71 | 0.12 | 0.25 | 0.22 |
| **Education** | | | | | |
| β coefficients | 0.05 | -0.01 | -0.02 | 0.61 | 0.77 |
| 95%CI | (-0.62,0.72) | (-0.04,0.02) | (-0.07,0.03) | (-0.14,1.36) | (-0.19,1.73) |
| *P*-Value | 0.25 | 0.89 | 0.74 | 0.41 | 0.28 |
| **Income** | | | | | |
| β coefficients | 0.001 | 0.001 | 0.002 | -0.003 | -0.004 |
| 95%CI | (-0.001,0.003) | (-0.002,0.004) | (-0.002,0.006) | (-0.007,0.001) | (-0.008,0.001) |
| *P*-Value | 0.33 | 0.24 | 0.29 | 0.25 | 0.21 |

PR: sperm progressive motility equals the spermatozoa moving actively, either linearly or in a large circle, regardless of speed.

NP: non-progressive motility equals all the other patterns of motility with an absence of progression.

^a^Result expressed as the relative percent change for volume, concentration, total sperm count. This number is converted from the antilog of the regression coefficient (β) of the log-linear model.

^b^For sperm progressive motility and total motility, result expressed as the absolute change.

^c^Multiple liner regression model was used for association of formaldehyde exposure with semen volume, concentration, total sperm count, sperm progressive motility and total motility, separately.

^d^Adjusted for age, cigarette intake, alcohol consumption, abstinence duration, body mass index, education, and income.

As potential confounders, age(years), BMI(kg/m^2^), duration of abstinence(days), and income (RMB/per month) were contin­uous variables, while cigarette intake (low, no smoking; moderate, <10 cigarettes/day; high, ≧10 cigarettes/day), alcohol consumption (low, no drinking; moderate, <1000g/day; high, ≧1000g/day), and education level (low, <6 years; high, ≧6 years) were categorical variables.

*** *P <* 0.05**

**Table 5--supplement** Associations of Formaldehyde exposure index (FEI) with semen kinematic parameters before and after adjusting for confounding factors ^a^

|  | VCL(μm/s) | VSL(μm/s) | LIN(VCL/VSL) | VAP(μm/s) | STR(VSL/VAP) | MAD(°) | ALH(μm) |
| --- | --- | --- | --- | --- | --- | --- | --- |
| **Crude β coefficients** | -0.07 | -0.05 | 0.001 | -0.05 | 0.002 | -0.01 | -0.003 |
| 95%CI | (-0.15,0.03) | (-0.12,0.02) | (-0.04,0.04) | (-0.12,0.02) | (-0.02,0.02) | (-0.05,0.03) | (-0.008,0.002) |
| *P*-Value | 0.27 | 0.31 | 0.95 | 0.39 | 0.98 | 0.55 | 0.22 |
| **Adjusted β coefficients ^b^** | -0.08 | -0.05 | 0.002 | -0.05 | 0.004 | -0.01 | -0.004 |
| 95%CI | (-0.18,0.04) | (-0.11,0.01) | (-0.04,0.04) | (-0.13,0.02) | (-0.02,0.03) | (-0.05,0.02) | (-0.010,0.001) |
| *P*-Value | 0.15 | 0.36 | 0.92 | 0.31 | 0.67 | 0.51 | 0.20 |
| **Age** | | | | | | | |
| β coefficients | -0.36 | -0.19 | 0.23 | -0.11 | 0.06 | 0.09 | -0.10 |
| 95%CI | (-0.79,0.07) | (-0.77,0.39) | (-0.16,0.61) | (-0.34,0.12) | (-0.16,0.28) | (-0.37,0.55) | (-0.17,-0.03) |
| *P*-Value | 0.08 | 0.33 | 0.25 | 0.64 | 0.61 | 0.70 | **0.03*** |
| **Cigarette intake** | | | | | | | |
| β coefficients | -0.35 | 0.58 | 0.22 | -1.07 | 1.36 | -1.71 | -0.36 |
| 95%CI | (-2.89,2.19) | (-5.43,6.59) | (-0.43,0.87) | (-3.68,1.54) | (0.28,2.44) | (-6.10,2.68) | (-0.92,0.21) |
| *P*-Value | 0.37 | 0.21 | 0.53 | 0.12 | **0.03*** | 0.13 | 0.22 |
| **Alcohol consumption** | | | | | | | |
| β coefficients | -0.23 | 0.95 | 0.28 | 0.25 | -0.75 | 0.20 | -0.38 |
| 95%CI | (-3.17,2.71) | (-3.93,5.83) | (-1.94,2.50) | (-1.62,2.12) | (-2.01,0.51) | (-2.46,2.86) | (-0.91,0.15) |
| *P*-Value | 0.88 | 0.15 | 0.80 | 0.23 | 0.24 | 0.88 | 0.13 |
| **Abstinence duration** | | | | | | | |
| β coefficients | 0.43 | 0.19 | 0.15 | -0.68 | 0.58 | 0.18 | -0.12 |
| 95%CI | (-0.72,1.58) | (-0.66,1.06) | (-0.70,0.99) | (-1.68,0.32) | (-0.20,1.36) | (-0.87,1.23) | (-0.26,0.02) |
| *P*-Value | 0.47 | 0.65 | 0.73 | 0.18 | 0.37 | 0.63 | 0.10 |
| **BMI** | | | | | | | |
| β coefficients | 0.17 | -0.58 | 0.51 | 0.22 | 0.31 | -0.14 | -0.08 |
| 95%CI | (-0.13,0.47) | (-0.17,1.33) | (-0.25,1.26) | (-0.81,1.24) | (-0.22,0.84) | (-1.54,1.26) | (-0.19,0.03) |
| *P*-Value | 0.54 | 0.13 | 0.19 | 0.47 | 0.14 | 0.56 | 0.51 |
| **Education** | | | | | | | |
| β coefficients | 0.36 | 0.16 | 0.24 | -0.82 | -0.13 | 1.62 | -0.07 |
| 95%CI | (-3.51,4.24) | (-2.69,3.01) | (-2.62,3.11) | (-4.19,2.54) | (-1.76,1.50) | (-1.81,5.06) | (-0.51,0.38) |
| *P*-Value | 0.85 | 0.91 | 0.87 | 0.63 | 0.87 | 0.35 | 0.76 |
| **Income** | | | | | | | |
| β coefficients | 0.002 | 0.001 | 0.001 | -0.001 | -0.001 | 0.001 | 0.001 |
| 95%CI | (-0.001,0.004) | (-0.001,0.003) | (-0.001,0.003) | (-0.004,0.002) | (-0.003,0.001) | (-0.002,0.004) | (-0.001,0.003) |
| *P*-Value | 0.32 | 0.40 | 0.36 | 0.46 | 0.27 | 0.32 | 0.42 |

^a^ Multiple liner regression model was used for association of formaldehyde exposure with sperm VCL, VSL, LIN, VAP, STR, MAD and ALH, separately.

^b^Adjusted for age, cigarette intake, alcohol consumption, abstinence duration, body mass index, education, and income.

As potential confounders, age(years), BMI(kg/m^2^), duration of abstinence(days), and income (RMB/per month) were contin­uous variables, while cigarette intake (low, no smoking; moderate, <10 cigarettes/day; high, ≧10 cigarettes/day), alcohol consumption (low, no drinking; moderate, <1000g/day; high, ≧1000g/day), and education level (low, <6 years; high, ≧6 years) were categorical variables.

*** *P <* 0.05**

**Table 6--supplement** ORs and 95%CI for below-normal values of semen parameters associated with FA occupational exposure before and after adjusting for confounding factors ^a^

|  | Semen volume (<1.5ml) | Sperm concentration  (<15×10^6^/ml) | Total sperm count  (<39×10^6^) | Sperm progressive motility (PR,%<32%) | Total motility (PR+NP,%<40%) |
| --- | --- | --- | --- | --- | --- |
| **Low-FA-exposed group (n=57) ^b^** | | | | | |
| **Crude OR** | 2.10 | 1.84 | 1.67 | 2.67 | 3.32 |
| 95%CI | (0.75,5.90) | (0.39,8.55) | (0.48,5.77) | (1.17,6.10) | (1.31,8.43) |
| *P*-Value | 0.16 | 0.44 | 0.42 | **0.02*** | **0.01*** |
| **Adjusted OR ^c^** | 1.83 | 1.67 | 1.59 | 2.58 | 3.21 |
| 95%CI | (0.63,5.36) | (0.33,8.43) | (0.45,5.61) | (1.11,5.97) | (1.24,8.28) |
| *P*-Value | 0.27 | 0.54 | 0.47 | **0.03*** | **0.02*** |
| **Age** |  |  |  |  |  |
| OR | 1.02 | 0.64 | 1.16 | 1.63 | 1.45 |
| 95%CI | (0.86,1.19) | (0.13,3.21) | (0.67,1.81) | (0.92,2.77) | (0.81,2.59) |
| *P*-Value | 0.86 | 0.58 | 0.35 | 0.12 | 0.19 |
| **Cigarette intake** |  |  |  |  |  |
| OR | 1.12 | 1.56 | 1.67 | 1.41 | 1.73 |
| 95%CI | (0.34,3.68) | (0.26,9.18) | (0.78,7.35) | (0.66,2.98) | (0.74,4.01) |
| *P*-Value | 0.13 | 0.41 | 0.15 | 0.37 | 0.21 |
| **Alcohol consumption** |  |  |  |  |  |
| OR | 1.83 | 1.21 | 1.13 | 0.64 | 0.46 |
| 95%CI | (0.76,4.44) | (0.72,2.02) | (0.39,3.24) | (0.29,1.49) | (0.16,1.30) |
| *P*-Value | 0.18 | 0.47 | 0.63 | 0.31 | 0.08 |
| **Abstinence duration** |  |  |  |  |  |
| OR | 0.87 | 0.72 | 0.91 | 1.27 | 2.16 |
| 95%CI | (0.58,1.16) | (0.19,2.74) | (0.61,1.33) | (0.85,1.71) | (1.15,4.04) |
| *P*-Value | 0.21 | 0.32 | 0.58 | 0.31 | **0.02*** |
| **BMI** |  |  |  |  |  |
| OR | 1.23 | 1.42 | 1.04 | 0.89 | 0.84 |
| 95%CI | (0.86,1.77) | (0.81,2.48) | (0.68,1.58) | (0.67,1.20) | (0.61,1.15) |
| *P*-Value | 0.26 | 0.22 | 0.87 | 0.54 | 0.26 |
| **Education** |  |  |  |  |  |
| OR | 1.02 | 1.01 | 1.07 | 0.96 | 1.01 |
| 95%CI | (0.32,3.32) | (0.88,1.15) | (0.25,4.64) | (0.37,2.51) | (0.73,1.41) |
| *P*-Value | 0.93 | 0.95 | 0.91 | 0.83 | 0.94 |
| **Income** |  |  |  |  |  |
| OR | 1.01 | 1.01 | 1.01 | 0.99 | 0.99 |
| 95%CI | (0.91,1.11) | (0.95,1.08) | (0.92,1.12) | (0.97,1.01) | (0.96,1.02) |
| *P*-Value | 0.92 | 0.85 | 0.89 | 0.94 | 0.94 |
| **High-FA-exposed group (n=57) ^b^** | | | | | |
| **Crude OR** | 2.63 | 1.35 | 2.32 | 3.88 | 4.96 |
| 95%CI | (0.96,7.18) | (0.26,6.96) | (0.72,7.51) | (1.73,8.72) | (2.00,12.31) |
| *P*-Value | 0.06 | 0.72 | 0.16 | **0.001*** | **0.001*** |
| **Adjusted OR^b^** | 2.28 | 1.25 | 1.73 | 3.41 | 4.84 |
| 95%CI | (0.75,6.91) | (0.21,7.35) | (0.49,6.15) | (1.45,7.92) | (1.83,12.81) |
| *P*-Value | 0.15 | 0.81 | 0.18 | **0.005*** | **0.001*** |
| **Age** |  |  |  |  |  |
| OR | 1.07 | 0.96 | 1.74 | 1.17 | 1.06 |
| 95%CI | (0.83,1.43) | (0.76,1.22) | (1.12,2.94) | (0.91,1.78) | (0.81,1.34) |
| *P*-Value | 0.66 | 0.75 | **0.03*** | 0.26 | 0.35 |
| **Cigarette intake** |  |  |  |  |  |
| OR | 1.07 | 1.78 | 1.36 | 1.14 | 1.34 |
| 95%CI | (0.12,2.82) | (0.23,8.28) | (0.62,5.20) | (0.55,2.35) | (0.63,3.56) |
| *P*-Value | 0.27 | 0.15 | 0.20 | 0.63 | 0.37 |
| **Alcohol consumption** |  |  |  |  |  |
| OR | 1.89 | 1.34 | 1.11 | 0.56 | 0.51 |
| 95%CI | (0.76,4.72) | (0.84,2.21) | (0.37,3.21) | (0.23,1.36) | (0.19,1.28) |
| *P*-Value | 0.17 | 0.34 | 0.65 | 0.20 | 0.12 |
| **Abstinence duration** |  |  |  |  |  |
| OR | 0.59 | 0.76 | 0.88 | 1.14 | 1.35 |
| 95%CI | (0.37,0.88) | (0.16,3.67) | (0.57,1.28) | (0.81,1.46) | (0.83,2.81) |
| *P*-Value | **0.04*** | 0.44 | 0.56 | 0.44 | 0.27 |
| **BMI** |  |  |  |  |  |
| OR | 1.01 | 1.27 | 1.20 | 0.96 | 0.88 |
| 95%CI | (0.71,1.38) | (0.73,2.22) | (0.82,1.78) | (0.35,2.66) | (0.66,1.21) |
| *P*-Value | 0.95 | 0.40 | 0.45 | 0.75 | 0.39 |
| **Education** |  |  |  |  |  |
| OR | 1.24 | 1.41 | 1.37 | 1.02 | 0.95 |
| 95%CI | (0.54,4.25) | (0.21,7.71) | (0.32,5.91) | (0.79,1.31) | (0.73,1.26) |
| *P*-Value | 0.57 | 0.44 | 0.51 | 0.87 | 0.81 |
| **Income** |  |  |  |  |  |
| OR | 1.02 | 0.99 | 1.01 | 1.01 | 1.01 |
| 95%CI | (0.85,1.21) | (0.96,1.03) | (0.97,1.05) | (0.95,1.08) | (0.96,1.06) |
| *P*-Value | 0.78 | 0.89 | 0.85 | 0.82 | 0.85 |

OR: odds ratio; CI: confidence interval. PR: sperm progressive motility equals the spermatozoa moving actively, either linearly or in a large circle, regardless of speed. NP: non-progressive motility equals all the other patterns of motility with an absence of progression.

Note: abnormal semen parameters were defined by the World Health Organization (WHO, 2010) standards: semen volume <1.5ml, sperm concentration < 15×10^6^/ml, total sperm count < 39×10^6^/ml, sperm progressive motility < 32% and total motility < 40%.

^a^ Logistic regression model was used for the association of formaldehyde exposure with below-normal values of semen volume, concentration, total sperm count, sperm progressive motility and total motility, separately.

^b^ compared with the reference group.

^c^ Adjusted for age, cigarette intake, alcohol consumption, abstinence duration, body mass index, education, and income.

As potential confounders, age(years), BMI(kg/m^2^), duration of abstinence(days), and income (RMB/per month) were contin­uous variables, while cigarette intake (low, no smoking; moderate, <10 cigarettes/day; high, ≧10 cigarettes/day), alcohol consumption (low, no drinking; moderate, <1000g/day; high, ≧1000g/day), and education level (low, <6 years; high, ≧6 years) were categorical variables.

*** *P <* 0.05**
